# Constitutive inhibitory G protein activity upon adenylyl cyclase-dependent cardiac contractility is limited to adenylyl cyclase type 6

**DOI:** 10.1371/journal.pone.0218110

**Published:** 2019-06-07

**Authors:** Caroline Bull Melsom, Marie-Victoire Cosson, Øivind Ørstavik, Ngai Chin Lai, H. Kirk Hammond, Jan-Bjørn Osnes, Tor Skomedal, Viacheslav Nikolaev, Finn Olav Levy, Kurt Allen Krobert

**Affiliations:** 1 Department of Pharmacology and Center for Heart Failure Research, Faculty of Medicine, University of Oslo and Oslo University Hospital, Oslo, Norway; 2 Department of Veterans Affairs, San Diego Healthcare System, San Diego, California, United States of America; 3 Department of Medicine, University of California, San Diego, California, United States of America; 4 University Medical Center Hamburg-Eppendorf, Hamburg, Germany; University of Torino, ITALY

## Abstract

**Purpose:**

We previously reported that inhibitory G protein (G_i_) exerts intrinsic receptor-independent inhibitory activity upon adenylyl cyclase (AC) that regulates contractile force in rat ventricle. The two major subtypes of AC in the heart are AC5 and AC6. The aim of this study was to determine if this intrinsic G_i_ inhibition regulating contractile force is AC subtype selective.

**Methods:**

Wild-type (WT), AC5 knockout (AC5KO) and AC6 knockout (AC6KO) mice were injected with pertussis toxin (PTX) to inactivate G_i_ or saline (control).Three days after injection, we evaluated the effect of simultaneous inhibition of phosphodiesterases (PDE) 3 and 4 with cilostamide and rolipram respectively upon *in vivo* and *ex vivo* left ventricular (LV) contractile function. Also, changes in the level of cAMP were measured in left ventricular homogenates and at the membrane surface in cardiomyocytes obtained from the same mouse strains expressing the cAMP sensor pmEPAC1 using fluorescence resonance energy transfer (FRET).

**Results:**

Simultaneous PDE3 and PDE4 inhibition increased *in vivo* and *ex vivo* rate of LV contractility only in PTX-treated WT and AC5KO mice but not in saline-treated controls. Likewise, Simultaneous PDE3 and PDE4 inhibition elevated total cAMP levels in PTX-treated WT and AC5KO mice compared to saline-treated controls. In contrast, simultaneous PDE3 and PDE4 inhibition did not increase *in vivo* or *ex vivo* rate of LV contractility or cAMP levels in PTX-treated AC6KO mice compared to saline-treated controls. Using FRET analysis, an increase of cAMP level was detected at the membrane of cardiomyocytes after simultaneous PDE3 and PDE4 inhibition in WT and AC5KO but not AC6KO. These FRET data are consistent with the functional data indicating that AC6 activity and PTX inhibition of G_i_ is necessary for simultaneous inhibition of PDE3 and PDE4 to elicit an increase in contractility.

**Conclusions:**

Together, these data suggest that AC6 is tightly regulated by intrinsic receptor-independent G_i_ activity, thus providing a mechanism for maintaining low basal cAMP levels in the functional compartment that regulates contractility.

## Introduction

Adenylyl cyclase (AC) is an important enzyme responsible for the synthesis of cAMP from ATP [1]. cAMP activates protein kinase A (PKA) which in turn phosphorylates a number of key Ca^2+^-cycling and -regulating proteins in the cardiomyocytes including L-type Ca^2+^ channels, ryanodine receptors, phospholamban and troponin-I. These effects produce increased and shorter cytoplasmic Ca^2+^ transients that increase the contractile response as well as hasten relaxation [2]. All AC isoforms except AC8 are detected in the heart at the transcript level [3, 4]; however AC5 and AC6 are the main isoforms responsible for cAMP synthesis in cardiomyocytes [5, 6]. Both AC5 and AC6 are activated by stimulatory G protein (G_s_) and inhibited by all three isoforms of inhibitory G protein (G_i_), with G_i_ inhibition being most effective at low levels of G_s_ activation [7]. In addition, both AC isoforms are inhibited by PKA phosphorylation [8, 9] and submicromolar concentrations of free Ca^2+^ [10], which may have important physiological implications in generating fluctuating Ca^2+^ and cAMP levels [11].

Despite the fact that AC5 and AC6 share ~65% amino acid sequence homology and many regulatory properties [5, 12], there are several important differences as to how their activity is regulated. AC5 is stimulated by PKCα and ζ [13], whereas AC6 is inhibited by PKCδ and ε [14, 15]. Further, basal activity of AC5 but not AC6 expressed in Sf9 cell membranes is inhibited by GTPγS-activated G_i_ [7]. AC5 localizes mainly in the t-tubular region of the cardiomyocytes where it is primarily associated with the β_2_-adrenergic receptor (β_2_AR) and is under tight restraint by phosphodiesterases (PDEs), whereas AC6 appears localized outside the t-tubules with the β_1_AR [16].

Previously, we have reported that after pertussis toxin (PTX) treatment to inactivate G_i_, combined PDE3 and PDE4 inhibition increases basal cAMP levels and evokes a large inotropic response in rat cardiac ventricular muscle strips [17–19]. In addition, PTX treatment enhanced the inotropic response to serotonin [20], forskolin, the G_s_-selective β_2_AR agonist (RR)-fenoterol [19] as well as generalized stimulation of β_1_- and β_2_ARs [18]. Taken together, these data suggest that G_i_ exerts a constant intrinsic inhibition upon AC independent of receptor activation. We propose that inactivation of G_i_ by PTX treatment shifts the balance of intrinsic G_i_ and G_s_ activity upon AC towards G_s_, enhancing the effect of all cAMP-mediated inotropic agents. To further understand the mechanism behind these findings, we wanted to investigate if the effects of receptor-independent intrinsic G_i_ inhibition were selective for either AC5 or AC6. To this end, we utilized AC5 and AC6 knockout mice and our data indicate that the effect of intrinsic G_i_ inhibition appears to be selective for AC6 in a compartment that regulates the contractile response.

## Methods

### Animal care

Animal use and care was approved by the Institutional Animal Care and Use Committee (IACUC) of the VA San Diego Healthcare System and the Norwegian Animal Research Authority and in accordance with National Institutes of Health guidelines and the European Convention for the protection of vertebrate animals used for experimental and other scientific purposes (Council of Europe no. 123, Strasbourg 1985). The animals were housed with a 12/12h cycle at 21°C, food and water available *ad libitum*.

### Animals

We acquired male and female heterozygote mice derived from a colony of either AC5-deficient (AC5KO) [21] or AC6-deficient (AC6KO) [22] mice. The original AC5KO had seven generations of backcross with C57Bl/6 [21]. The original AC6KO had ten generations of backcross with C57Bl/6. The two colonies from which our animals were obtained were kept by crossing the respective heterozygotes. Upon receipt of the heterozygotes from these two colonies, we bred the respective AC5 or AC6 heterozygotes producing hybrids of either homozygote (ACx^-/-^) (KO), heterozygote (ACx^+/-^) and WT (ACx^+/+^) littermates. When a sufficient number of AC5^-/-^, AC6^-/-^ and WT were obtained, thereafter, we bred the AC5^-/-^ or AC6^-/-^ or WT littermates respectively to maintain colonies of each strain. Two to six month old male or female mice bred from these colonies were used for experimentation. To verify the integrity of each strain, genotype analyses were performed using PCR with specific primers for the mutant and wild type alleles as previously described [21, 22].

Transgenic mice used for the FRET experiments were made by crossing transgenic pmEPAC1 FVB/N mice [23] with our WT, AC5KO or AC6KO mice. Those offspring expressing pmEPAC1 (now heterozygous for the respective AC and pmEPAC1 gene) were bred with either AC5KO or AC6KO mice until AC5KO or AC6KO homozygotes were obtained containing the pmEPAC1 sensor. Thereafter, the homozygous AC5KO or AC6KO mice containing the pmEPAC1 sensor were bred together to obtain mice used for experimentation. All offspring were genotyped for ACKO as described above and for the pmEPAC1 sensor gene as described previously [23].

### PTX treatment

PTX (Merck chemicals, Nottingham, UK) was administered at a dose of 60 μg/kg i.p. (*in vivo* studies) or 120 μg/kg i.p. (*ex vivo* and cAMP studies) as a single injection three days prior to study. Control mice were given a saline injection of equal volume. To confirm the effectiveness of PTX, the level of PTX-catalyzed incorporation of [^32^P]ADP-ribose from [^32^P]NAD (PerkinElmer, Boston, MA) into available G_i_ was measured as previously described [17]. Data from animals treated with PTX were only included if carbachol did not reverse the cilostamide and rolipram-evoked inotropic.

### Left ventricular *in vivo* contractile function

Mice were anesthetized with 5% induction and 2% maintenance isoflurane (Forene, Baxter Pharmaceutics, Deerfield, IL). A 1F or 1.4F conductance-micromanometer catheter (Millar Instruments, Houston, Texas) was inserted via the right carotid artery across the aortic valve and into the left ventricular (LV) chamber. Bilateral vagotomy was performed to minimize confounding effects of autonomic reflex activation. LV maximal pressure development (+(dP/dt)_max_) and maximal rate of pressure decline (-(dP/dt)_max_; as an index of diastolic function), given as absolute values, thus max indicates the maximum negative value) as well as heart rate were recorded. Mice were allowed to stabilize prior to recording baseline. Timolol (timolol maleate, Sigma-Aldrich, St. Louis, MO, USA; 2.5 mg/kg i.p.), a non-selective βAR antagonist with inverse agonistic properties, was administered to eliminate the effect of endogenously activated or constitutively active βARs. Subsequently, we measured the effect of the PDE3 inhibitor cilostamide (Tocris Bioscience, Bristol, UK; 3 mg/kg i.p.) and the PDE4 inhibitor rolipram (Tocris Bioscience; 10 mg/kg i.p) in combination upon heart contractility. To verify continuous and complete βAR blockade, timolol (2.5 mg/kg i.p.) was administered a second time after the PDE inhibitors. This was done to control that the responses to PDE inhibition did not result from PDE potentiation of residual endogenous adrenergic stimulation. Drug interventions were given 4 minutes apart and animals were sacrificed at the end of the experiment. LV was flash-frozen for the ADP-ribosylation assay to verify the effectiveness of PTX.

### Left ventricular *ex vivo* contractile function

LV strips (~1 mm diameter) were prepared from WT, AC5KO and AC6KO mice in a 0.2 mM Ca^2+^ buffer containing 20 mM 2,3-butanedione monoxime (BDM) [24, 25]. After isolation, the strips were mounted in 31°C organ baths containing 0.2 mM Ca^2+^ buffer containing 20mM BDM and stretched until ~0.2–0.3 grams of basal tension. After 5–15 minutes, the organ bath solution was changed to a physiological salt solution with 1.8 mM Ca^2+^, equilibrated and field-stimulated at 1 Hz as previously described [24, 25]. Contraction-relaxation cycles were recorded and analyzed as previously described [26, 27]. Maximal development of force (dF/dt)_max_ was measured and inotropic responses were expressed as percent increase above basal. Lusitropic responses were expressed as changes in relaxation time (time to 80% relaxation—time to peak force). The effect of combined PDE3 inhibition (cilostamide, 1 μM) and PDE4 inhibition (rolipram, 10 μM) was assessed in the presence of the non-selective βAR antagonist timolol (1 μM) and α_1_-adrenergic receptor antagonist prazosin (0.1 μM). To assess the functional effectiveness of PTX treatment, a single 10 μM dose of the muscarinic agonist carbachol was given approximately 10–20 minutes after cilostamide and rolipram. This was followed by addition of atropine (1 μM) to reverse effects of carbachol. In addition, to assess the effectiveness of the prior timolol blockade, another 1 μM dose of timolol was given. The absence of timolol to reverse the inotropic response indicated that the cilostamide and rolipram-evoked inotropic response in PTX treated animals did not result from incomplete blockade of βARs. The experiment was concluded after administration of 100 μM isoproterenol to overcome the timolol blockade to verify the ability of the muscle to elicit an inotropic response.

### Measurement of cAMP level

In a subset of mice treated with or without PTX, LV strips were flash frozen and used to assess cAMP levels. LV strips were prepared as described above and treated with cilostamide (1 μM) and rolipram (10 μM) for ~20 minutes and then flash frozen in liquid nitrogen. Frozen strips were homogenized in 5% TCA (Trichloroacetic acid, Sigma-Aldrich) and cAMP levels were measured by radioimmunoassay as previously described [28]. Protein was measured with the Coomassie Plus Protein Assay (Pierce, Rockford, IL) according to the manufacturer’s protocol and cAMP levels were normalized to the amount of protein in each sample.

### Isolation of cardiomyocytes

The whole heart was rapidly excised from the anesthetized mouse and the aorta quickly cannulated on a Langendorff perfusion system for retrograde aorta perfusion. A Ca^2+^-free perfusion buffer (in mM: NaCl: 120.4; KCl: 14.7; KH_2_PO_4_: 0.6; Na_2_HPO_4_: 0.6; MgSO_4_-7H_2_O: 1.2; Na-HEPES buffer: 10; NaHCO_3_: 4.6; Taurine: 30; BDM: 10; Glucose: 5.5; pH 7.0) was first used for 4 min, followed by enzymatic digestion (about 22 min) using collagenase type 2 solution (310 u/mg) prepared at 1 mg/ml in perfusion buffer. Thereafter, ventricles were cut into small pieces and gently triturated in perfusion buffer. The buffer’s Ca^2+^ content was gradually increased (in 3 steps) from 0.2 mM to a final concentration of 1.2 mM (incubation for 5 min each) and supplemented with fetal bovine serum. In between each calcium reintroduction step, following sedimentation of the cardiomyocytes (5 min) they were centrifuged (17g for 3 min). After the last centrifugation, the pellet of cardiomyocytes were resuspended in plating medium (MEM 0.85g/l NaHCO_3_, BSA, penicillin/streptomycin, glutamine and BDM 10mM) and seeded on cover slides pre-coated with laminin. The cardiomyocytes were used for FRET experimentation ~5–24 hours after isolation.

### Fluorescence resonance energy transfer (FRET) imaging

FRET-based measurements were performed in isolated cardiomyocytes harvested from pmEPAC1-WT/AC5KO/AC6KO transgenic mice. Isolated cardiomyocytes were attached to acid-washed and laminin-coated 24 mm glass cover slides in an 8-well dish. One cover slide was placed in a watertight imaging chamber (Attofluor; Life technologies) at room temperature with FRET buffer (5.4 mM KCl, 144 mM NaCl, 1mM MgCl_2_, 10 mM HEPES, 1 mM CaCl_2_, pH 7.4). Cells were excited at 436±10 nm and 500±10 nm using a monochromator (Polychrome V: FEI Munich), and emission from CFP and YFP were separated using a Dichrotome iMIC Dual Emission Module where a 515 nm LP filter separated the images from CFP and YFP onto a single EM CCD camera chip (EVOLVE 512, Photometrix). Images were acquired by Live Acquisition browser (FEI Munich) and FRET was calculated using Offline Analysis (FEI Munich). FRET ratios were measured as ratios of YFP and CFP emission (F_530_/F_470_). YFP emission was corrected for direct excitations at 436 nm and spillover of CFP emission into the 530nm channel. The direct excitation of CFP at 500 nm and YFP emission at 470 nm was negligible. Cardiomyocytes were incubated for 3 hours with 1 μg/ml PTX or saline of equal volume in the incubation media (1.2 ml reaction volume). The FRET signal in response to simultaneous application of cilostamide (1 μM) and rolipram (10 μM) was measured in the presence of 1 μM timolol to block βARs. This was followed approximately five to seven minutes later by application of 100 μM forskolin which was regarded as giving the maximal FRET response. Only cells that responded to forskolin were included in the data set.

### Statistics

Data are expressed as mean ± SEM from n animals. P<0.05 was considered statistically significant (two-way ANOVA or two-way repeated measures ANOVA with Sidak’s or Tukey’s adjustment for multiple comparisons respectively and student’s t-test when appropriate).

## Results

### G_i_ inactivation by pertussis toxin

**Fig 1:**
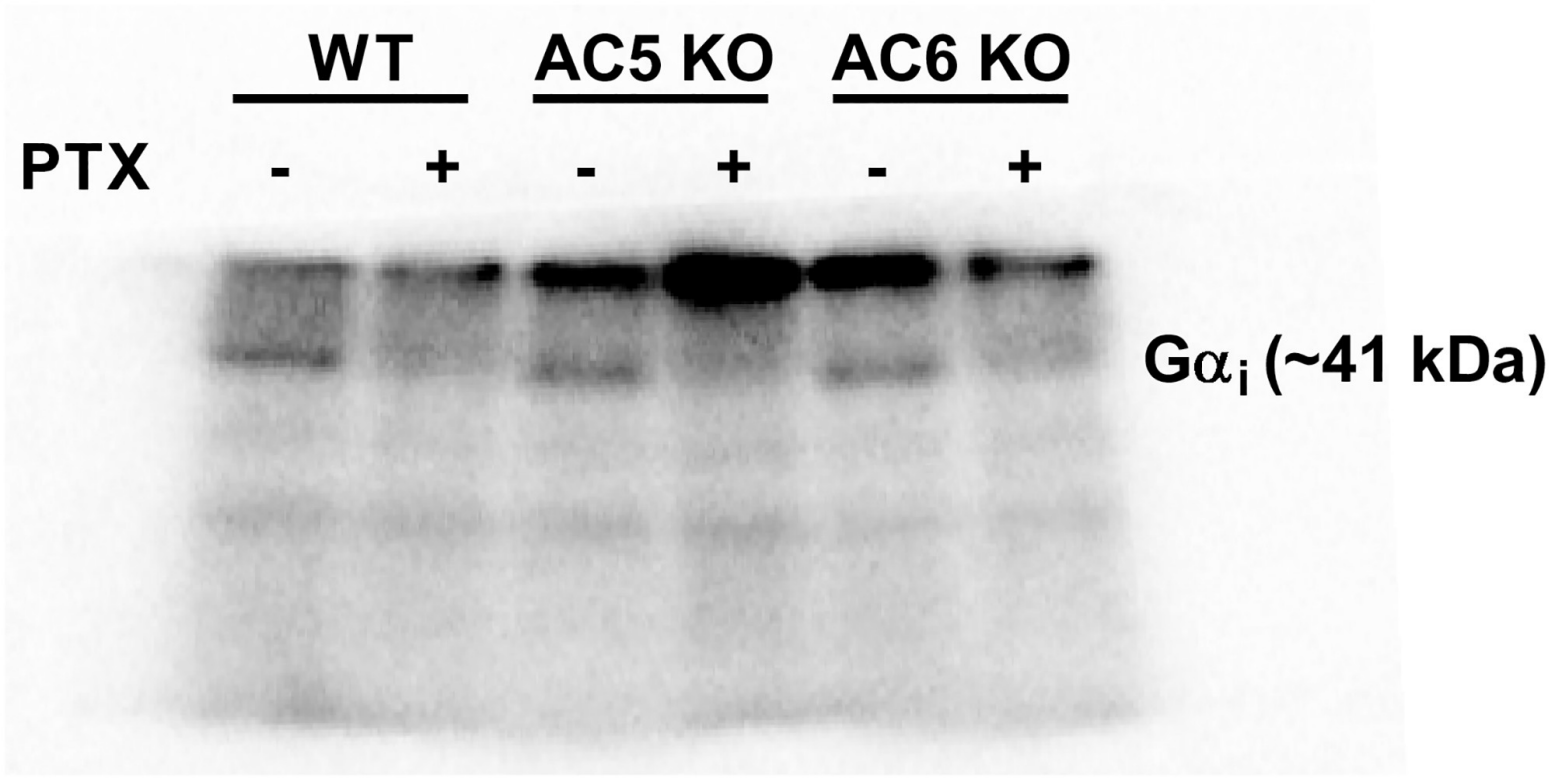
Representative autoradiogram showing ADP-ribosylated Gα_i_ protein in WT, AC5KO and AC6KO mouse left ventricle pre-treated with saline or pertussis toxin (PTX).

The effectiveness of *in vivo* PTX treatment to inhibit G_i_ was assessed by measuring PTX-catalysed incorporation of [^32^P]ADP-ribose from [^32^P]NAD into available G_i_ in a subset of each mouse strain (n = 6). An ~70% reduction was seen in the ability of subsequent PTX to ADP-ribosylate G_i_ *in vitro* in animals pre-treated with PTX compared to animals given saline injection (Fig 1). Animals were included in the *ex vivo* muscle studies, only when administration of the muscarinic agonist carbachol did not reverse the cilostamide/rolipram-evoked inotropic response in PTX treated mice (data not shown; see Methods section for inclusion criteria).

All mice received either saline or PTX injection (60 μg/kg i.p.) three days prior to study. The level of PTX-catalyzed incorporation of [^32^P]ADP-ribose from [^32^P]NAD into available G_i_ was measured as described in methods. Each sample loaded contained 140 μg of protein. To obtain the percentage of G_i_ ADP ribosylated in the PTX vs. saline injected mice, the optical density of each G_i_ band was measured and the effectiveness of the PTX treatment was calculated by dividing the optical density of the PTX-treated sample by the saline-treated sample in the respective adjacent lane. In animals pre-treated with PTX compared to animals given saline injection, there was a 69 ± 2, 64 ± 9 and 72 ± 4% reduction (in WT, AC5KO and AC6KO respectively) in the ability of subsequent PTX to ADP-ribosylate G_i_ in vitro. Data are mean ± SEM. n = 6 for all mouse strains.

### The increase in contractility observed after PDE3, PDE4 and G_i_ inhibition is absent in AC6 KO mice

**Fig 2:**
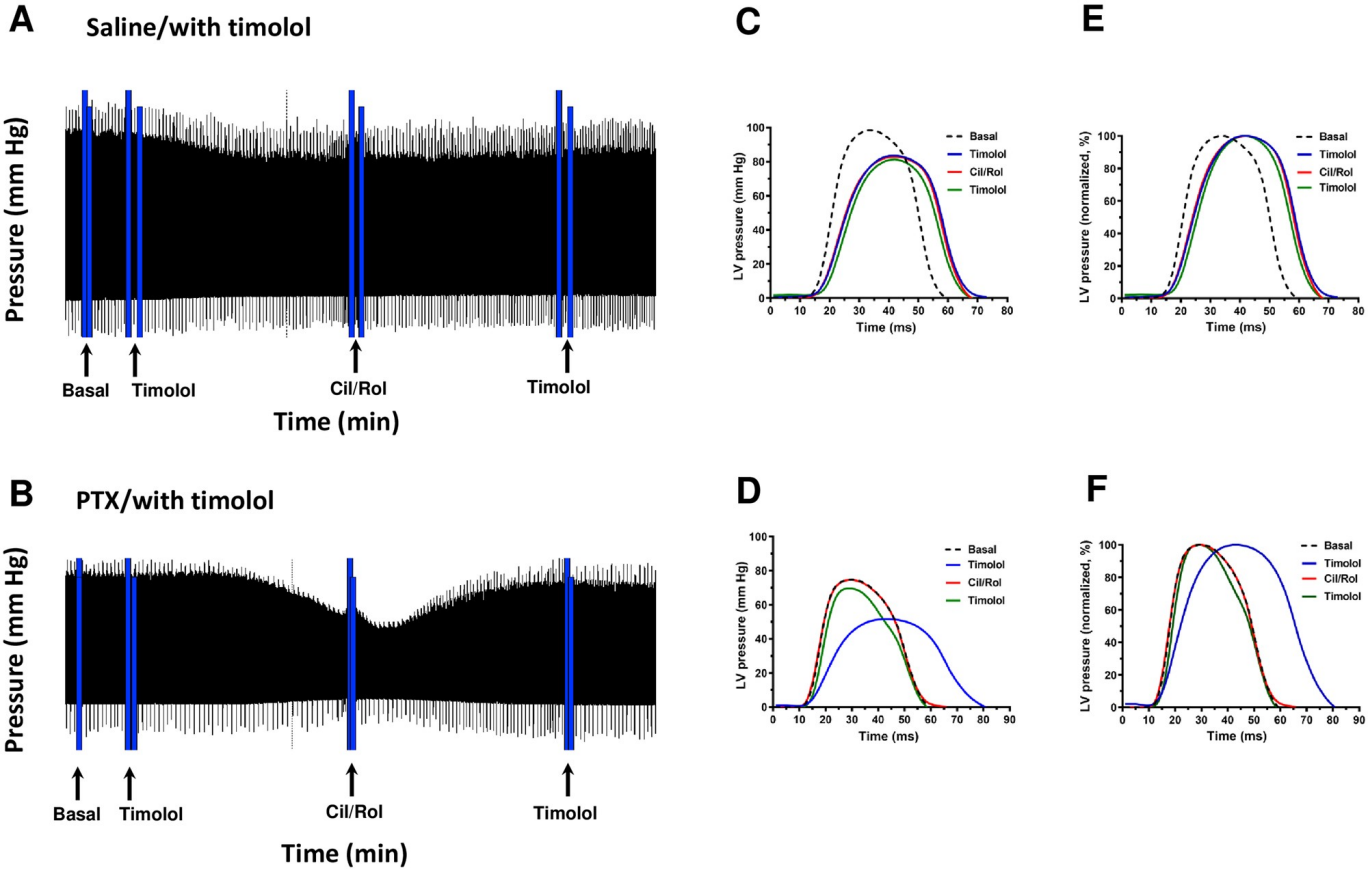
Experimental protocol and analysis of *in vivo* experiments. (A,B) Representative screenshots from wild-type mice showing left ventricular pressure (mm Hg) over time during baseline, followed by 1 μM timolol, followed by the response to simultaneous addition of 1 μM cilostamide (Cil) and 10 μM rolipram (Rol) and lastly a second administration of timolol to verify responses to cilostamide and rolipram did not result from βAR activation. Displayed is the continuous recording of left ventricular pressure over the entire experimental period. The mice were pre-treated with either saline (A,C,E) or PTX (B,D,F; 120 μg/kg i.p. three days prior to study). Note that cilostamide and rolipram evoked an increase in pressure only in PTX treated mice in the presence of timolol. (C-F) Shown are individual contraction-relaxation cycles of changes in pressure from before and after addition of each respective drug administration expressed in mm Hg (C,D) or as a percentage of each individual maximal pressure (E,F; normalized to 100%) to illustrate the time base of the contraction-relaxation cycle. Cilostamide and rolipram increased pressure in AC5KO similar to WT but not in AC6KO (see Fig 3).

*In vivo* change in contractility was indirectly inferred by measuring pressure (mm Hg) in WT, AC5KO and AC6KO mice using a conductance-micromanometer catheter inserted into the LV. An increase in the maximal development of pressure (+(dP/dt)_max_) during systole served as an index of an increase in contractility, whereas during the diastolic phase the time course of -(dP/dt) and maximal rate of pressure decline (-(dP/dt)_max_) was taken to reflect diastolic function. The heart rate was also recorded. We measured the response to inhibition of cAMP degradation using a combination of PDE3 (cilostamide; 3 μg/kg i.p.) and PDE4 (rolipram; 10 μg/kg i.p.) inhibitors in animals pre-treated with PTX or saline three days prior (Fig 2).

**Fig 3:**
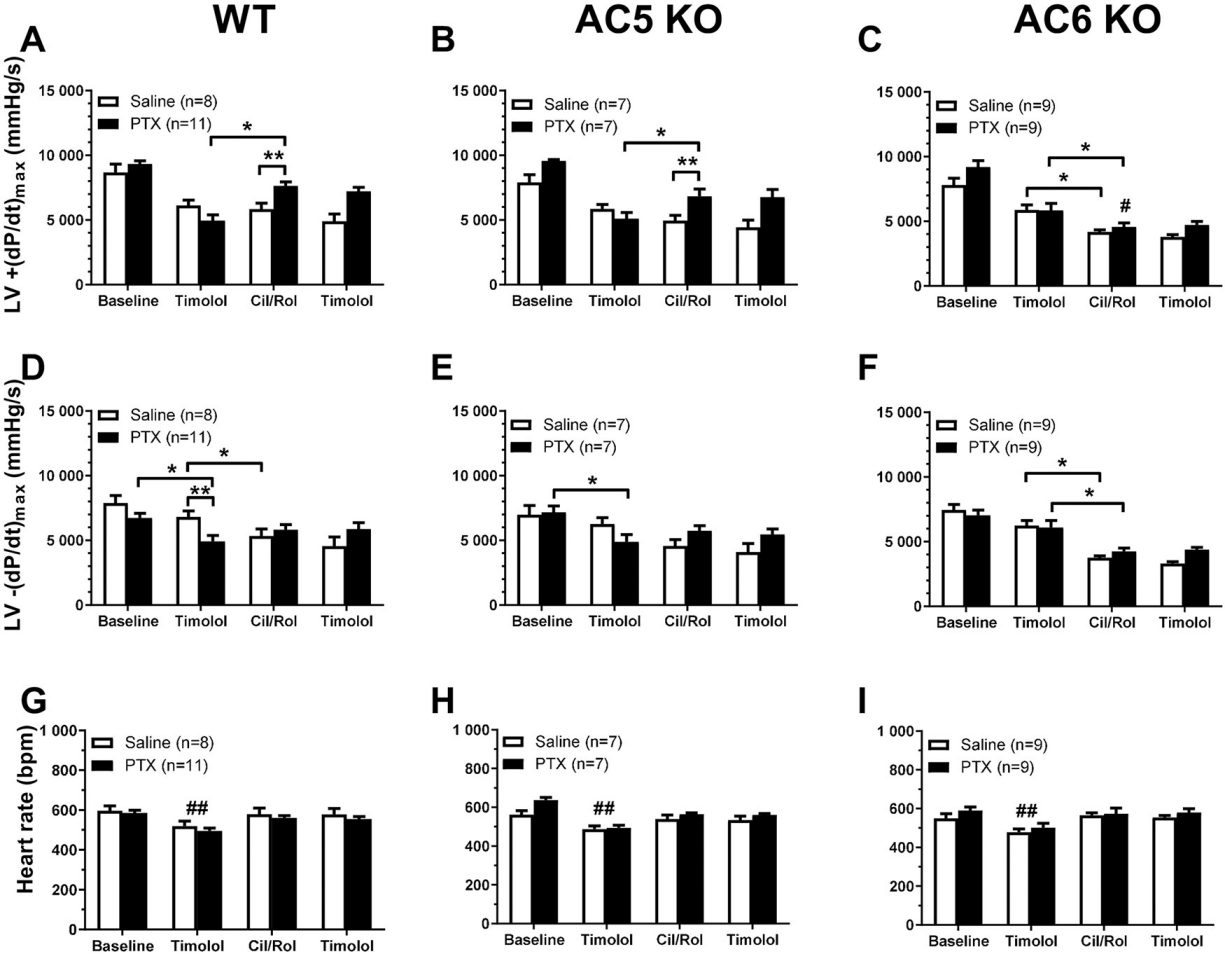
PDE3 and PDE4 inhibition after PTX treatment increased *in vivo* rate of left ventricle contractility in wild type (WT) and AC5KO mice but not in AC6KO mice. Left ventricular (LV) +(dP/dt)_max_ (A-C), -(dP/dt)_max_ (D-F) and heart rate (G-I) recorded in WT (A, D, G), AC5KO (B, E, H) and AC6KO (C, F, I) mice treated first with timolol (2.5 mg/kg i.p.) followed by combined PDE3 inhibitor cilostamide (3 mg/kg i.p.) and PDE4 inhibitor rolipram (10 mg/kg i.p.). To verify continuous and complete βAR blockade, timolol (2.5 mg/kg i.p.) was administered a second time after the PDE inhibitors. All mice received either saline or PTX injection i.p. three days prior to study. Experiments were conducted after vagotomy. Data are mean±SEM. *P<0.05, two-way repeated measures ANOVA with Tukey’s multiple comparisons test; **P<0.05, two-way ANOVA with Sidak’s multiple comparisons test; #P<0.05 PTX-treated AC6KO vs. PTX-treated WT and AC5KO, two-way ANOVA with Sidak’s multiple comparisons test. ##P<0.05 vs. baseline, Cil/Rol and the second timolol in respective saline and PTX treated groups, two-way repeated measures ANOVA with Tukey’s multiple comparisons test.

Baseline +(dP/dt)_max_, baseline -(dP/dt)_max_ and heart rate were similar for the different mouse strains (in both saline and PTX-treated) and after PTX treatment in any strain (Fig 3A–3I). The first administration of timolol reduced the +(dP/dt)_max_ compared to baseline in all three strains of mice by ~20–30% in saline-treated animals and ~35–45% in PTX-treated animals (P<0.05, two-way repeated measures ANOVA with Tukey’s multiple comparisons test, Fig 3A–3C) but values were similar across the strains or by PTX treatment. Timolol treatment also reduced -(dP/dt)_max_ compared to baseline in all three strains of mice by ~10–16% in saline- and ~13–32% in PTX-treated animals (P<0.05, two-way repeated measures ANOVA with Tukey’s multiple comparisons test, Fig 3D–3F), which did not differ between the mouse strains for either saline or PTX-treated groups. Timolol reduced -(dP/dt)_max_ more in PTX-treated than saline-treated in WT and AC5KO, but not in AC6KO (Fig 3D–3F). Timolol also decreased heart rate ~15–20% compared to baseline (Fig 3G–3I) in all three mouse strains with similar values for all mouse strains or PTX treatment groups. That timolol reduced +(dP/dt)_max_, -(dP/dt)_max_ and heart rate are consistent with the fact that contractile function of mouse heart is highly dependent upon sympathetic activation of βARs.

After timolol treatment, combined PDE3 and PDE4 inhibition by cilostamide and rolipram respectively evoked an increase in +(dP/dt)_max_ in PTX-treated WT and AC5KO mice (~54% and ~34% respectively, Fig 3A and 3B) but not in saline-treated WT or AC5KO. In stark contrast to WT and AC5KO, cilostamide and rolipram decreased +(dP/dt)_max_ in both saline and PTX-treated AC6KO (~29% and ~22% respectively, Fig 3C).

Cilostamide and rolipram decreased the -(dP/dt)_max_ compared to prior timolol values in saline-treated WT, AC5KO and AC6KO (Fig 3D–3F; Table 1). In PTX-treated mice, however, the -(dP/dt)_max_ was similar compared to prior timolol values in WT and AC5KO (Fig 3D and 3E), but decreased in AC6KO (Fig 3F). To further quantify this qualitative difference produced by PTX treatment, we calculated the effect of cilostamide and rolipram treatment after timolol on the -(dP/dt)_max_ values (Table 1). As shown in Table 1, combined cilostamide and rolipram treatment tended to increase the -(dP/dt)_max_ in PTX-treated WT and AC5KO mice as opposed to a decrease in respective saline-treated mice. In contrast, combined cilostamide and rolipram treatment decreased -(dP/dt)_max_ in both saline- and PTX-treated AC6KO mice (Table 1). As -(dP/dt)_max_ largely reflects +(dP/dt)_max_, an increase in -(dP/dt)_max_ is consistent with the increase in +(dP/dt)_max_ observed in PTX-treated WT and AC5KO mice compared to respective saline-treated controls (Fig 3A, 3B, 3D and 3E). Further, the -(dP/dt)_max_ was decreased after cilostamide and rolipram treatment by a similar magnitude in both saline and PTX-treated AC6KO mice (Fig 3F; Table 1). In summary, these data indicate that the cilostamide and rolipram-mediated increase in +(dP/dt)_max_ (inotropic effect) and -(dP/dt)_max_ (diastolic effect) observed only in PTX-treated mice are dependent on the presence of AC6.

**Table 1:** Effect of PDE3 and PDE4 inhibition on -(dP/dt)_max_.

	Saline-treated, mmHg/s (n)	PTX-treated, mmHg/s (n)
WT	-1491±467 (8)	890±534 (11)^a^
AC5KO	-1686±698 (7)	844±576 (7)^a^
AC6KO	-2488±357 (9)	-1656±439 (9)^b^

Shown are the differences (mean±SEM) between the -(dP/dt)_max_ recorded after timolol and after subsequent cilostamide and rolipram (Cil/Rol). Note that in PTX-treated mice, Cil/Rol increased the -(dP/dt)_max_ after timolol in WT and AC5KO but decreased it in AC6KO. In contrast, Cil/Rol decreased the -(dP/dt)_max_ in all groups that were saline-treated. Data are given in mmHg/s (mean±SEM).

^a^ P<0.05 Saline *vs* PTX in respective group, two-way ANOVA with Tukey’s multiple comparisons test;

^b^ P<0.05 AC6KO PTX *vs* WT PTX and AC5KO PTX, two-way ANOVA with Tukey’s multiple comparisons test.

In all three mouse strains (both saline and PTX-treated), the first timolol treatment decreased heart rate which returned to baseline levels after cilostamide and rolipram treatment (Fig 3G–3I). Heart rate did not differ across either the mouse strain or treatment (saline or PTX) throughout the experiment (Fig 3G–3I). Therefore, changes in contractility observed after combined cilostamide and rolipram treatment unlikely result from changes in heart rate. Importantly, timolol given after cilostamide and rolipram produced no response on either +(dP/dt)_max_, -(dP/dt)_max_ or heart rate. This indicates that the initial dose of timolol was sufficient to block any confounding effects mediated by activation of βARs (Fig 3A–3I).

### PDE3, PDE4 and G_i_ inhibition elicit an AC6 dependent inotropic response in left ventricular myocardium *ex vivo*

**Fig 4:**
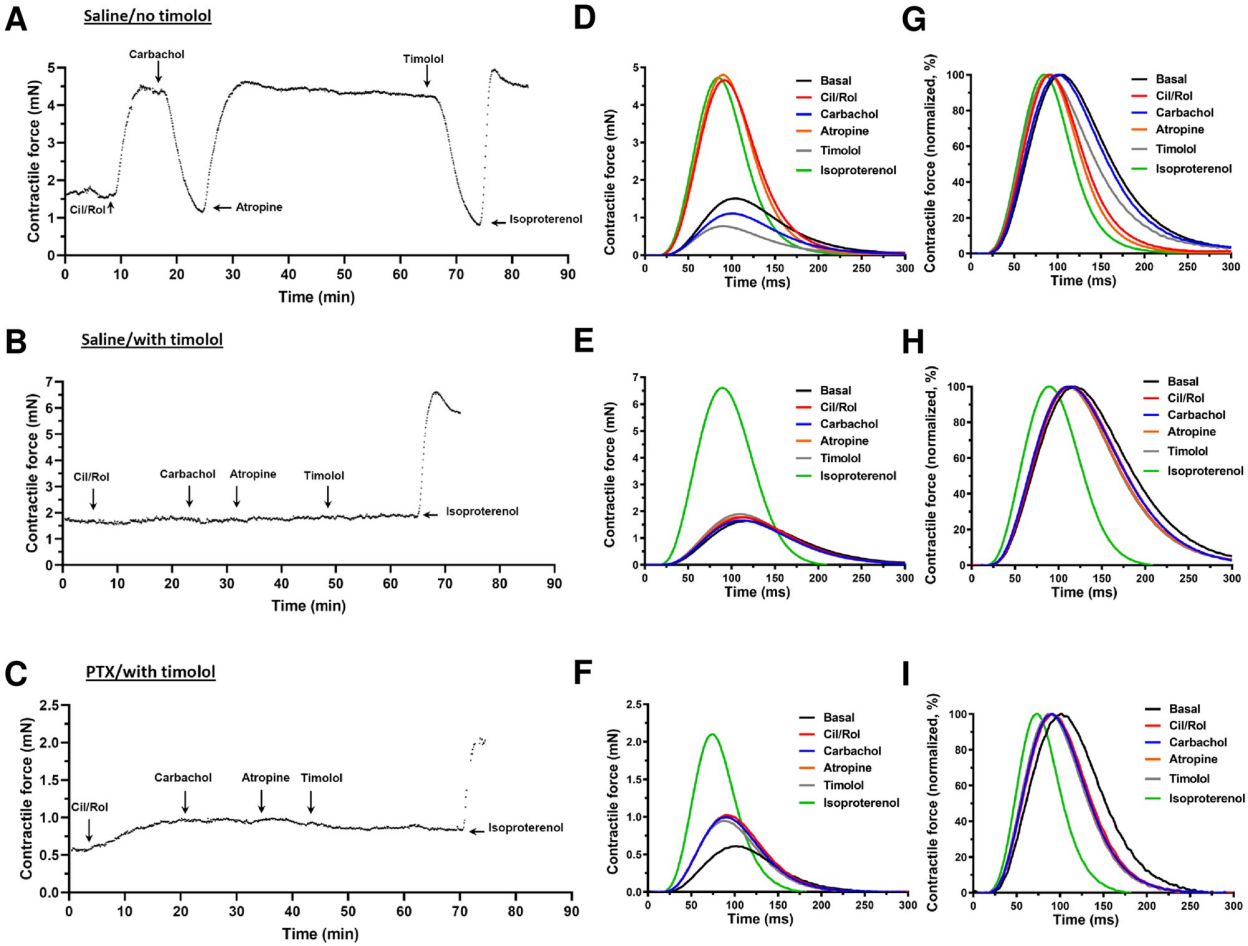
*Ex vivo* experimental timeline and contraction-relaxation cycles. (A-C) Representative traces showing inotropic responses (mN) evoked by simultaneous addition of 1 μM cilostamide (Cil) and 10 μM rolipram (Rol) and the subsequent effect of 10 μM carbachol and reversal of carbachol effects by 1 μM atropine followed by 1 μM timolol in left ventricular strips of WT mice. The mice were pre-treated with either saline (A,B) or PTX (C; 120 μg/kg i.p. three days prior to study). Displayed is the F_max_ of each contraction–relaxation cycle (CRC) in 1 ms intervals (each point) over the entire experimental period (A,B,C). Note that timolol blockade of βARs in animals pre-treated with saline was sufficient to prevent an inotropic response by cilostamide and rolipram. In addition, cilostamide or rolipram evoked an inotropic and lusitropic response only in PTX treated mice in the presence of timolol. (D-F) Averaged CRCs (mean data of 10–30 consecutive cycles) from before and after each drug administration expressed in mN or as (G-I) a percentage of each individual maximum F_max_ to illustrate lusitropic effects as reduction of relaxation time. Similar effects were obtained for AC5KO as WT, but cilostamide and rolipram elicited no inotropic or lusitropic effect in AC6KO (see Fig 5).

**Fig 5:**
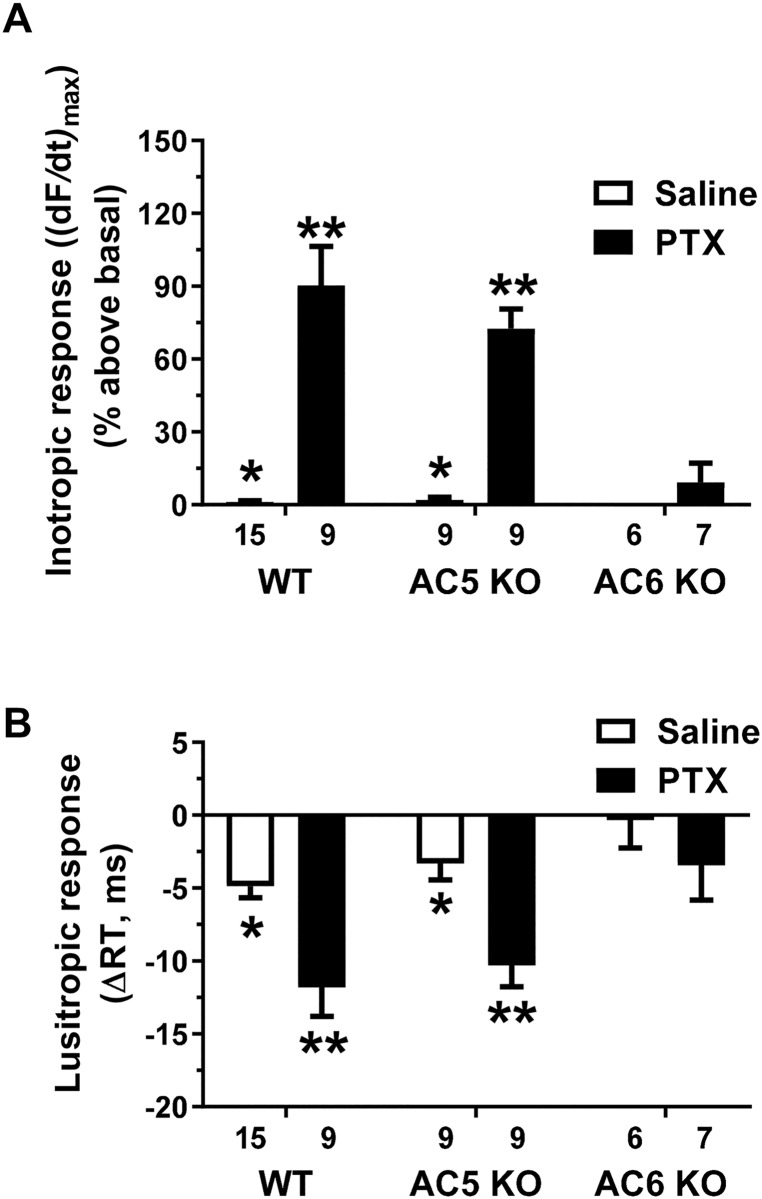
Knockout of AC6 prevented the inotropic and lusitropic response to combined PDE3 and PDE4 inhibition in isolated ventricular strips from mice treated with pertussis toxin (PTX). (A) Maximal inotropic response (reported as (dF/dt)_max_ in percent above basal) and (B) lusitropic response (reported as Δ relaxation time (RT) in ms) elicited by simultaneous addition of PDE3 (cilostamide, 1 μM) and PDE4 (rolipram, 10 μM) inhibitors in left ventricular strips from saline-treated or PTX-treated mice (120 μg/kg i.p. three days prior to study). All experiments were conducted in the presence of the βAR blocker timolol (1 μM), α_1_-adrenergic receptor antagonist prazosin (0.1 μM) followed by addition of cilostamide and rolipram. Data are mean±SEM. *P<0.05 vs. respective PTX treated group, two-way ANOVA with Sidak’s multiple comparisons test. **P<0.05 vs. PTX-treated AC6KO, two-way ANOVA with Tukey’s multiple comparison’s test.

We also measured the *ex vivo* inotropic response in LV strips from WT, AC5KO and AC6KO mice in the absence and presence of timolol (1μM), using a method directly measuring changes in contractile force adapted to mice as previously used for rat [24, 25]. In the absence of timolol, combined PDE3/PDE4 inhibition by simultaneous addition of cilostamide and rolipram elicited a large inotropic and lusitropic response in saline-treated animals of all three mouse strains (representative data shown only for WT, Fig 4A). However, in the presence of timolol, combined simultaneous addition of cilostamide and rolipram elicited no inotropic response in saline-treated animals of any strain (Figs 4B, 4E, 4H and 5A). This indicates that blockade of the βARs is adequate to prevent the cilostamide and rolipram-elicited inotropic response in saline treated animals. In contrast, after PTX treatment, simultaneous addition of cilostamide and rolipram evoked an inotropic response in WT and AC5KO but not in AC6KO (data shown only for WT in Fig 4C and 4F; Fig 5A). Likewise, the lusitropic response was larger (*i*.*e*. the relaxation time decreased more) after addition of cilostamide and rolipram in PTX-treated than saline-treated WT and AC5KO, and absent (i.e. relaxation time was unchanged) in both PTX- and saline-treated AC6KO (data shown only for WT in Fig 4H and 4I; Fig 5B).

### PDE3, PDE4 and G_i_ regulation of adenylyl cyclase activity is consistent with the functional effects

**Fig 6:**
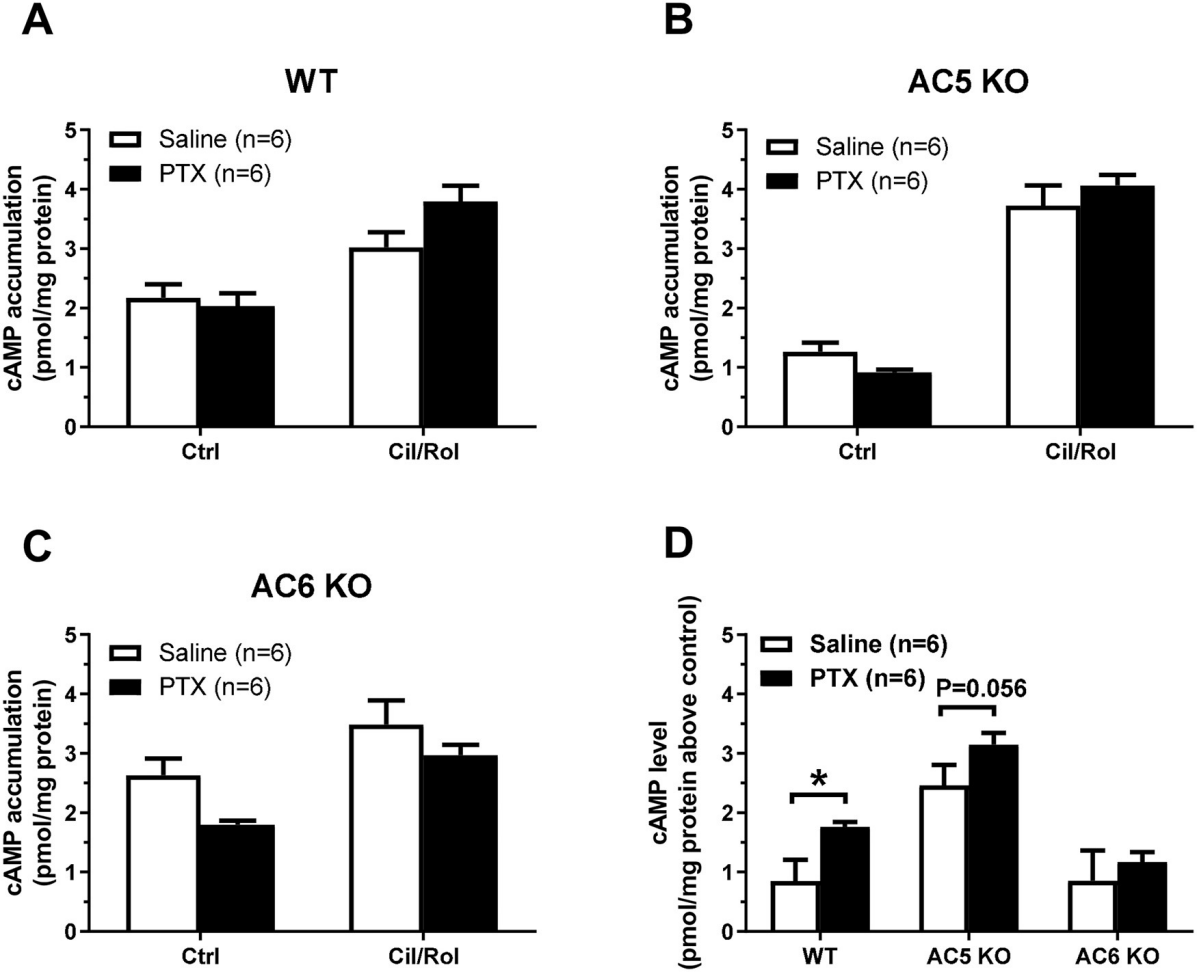
PTX treatment increased cAMP levels evoked by simultaneous inhibition of PDE3 and PDE4 is dependent on AC6. The effect of simultaneous inhibition of PDE3 (cilostamide, 1μM) and PDE4 (rolipram, 10 μM) upon cAMP levels in left ventricular homogenates after saline or PTX pre-treatment (120 μg/kg i.p. three days prior to study). The experiments were conducted in the presence of the βAR inverse agonist timolol (1 μM) and α_1_-adrenergic receptor antagonist prazosin (0.1 μM). The strips were flash frozen in liquid nitrogen after ~20 minutes of incubation with the PDE inhibitors. Data are mean±SEM and reported as pmol cAMP/mg protein (A,B,C) or pmol cAMP/mg protein above control (no cilostamide or rolipram) (D). *P<0.05, unpaired t-test.

Next, we measured the effect of combined cilostamide and rolipram treatment upon cAMP levels in homogenized LV muscle. Animals of each strain were pre-treated three days prior with either saline or PTX and two strips were excised from each heart with one given cilostamide and rolipram and the other serving as control without PDE inhibition. Basal cAMP levels were lower in both the saline- and PTX-treated AC5KO control compared to WT and AC6KO control (P<0.05, two-way ANOVA with Tukey’s multiple comparison’s test; Fig 6A–6C; basal levels of cAMP (pmol cAMP/mg protein): WT saline: 2.17±0.23; WT PTX: 2.03±0.22; AC5KO saline: 1.27±0.15; AC5KO PTX: 0.91±0.05; AC6KO saline: 2.63±0.28; AC6KO PTX: 1.80±0.07). Cilostamide and rolipram together increased the cAMP levels above control in all six conditions (~1–3 pmol/mg protein), and more in AC5KO than in WT and AC6KO (P<0.05, main effect of mouse strain two-way ANOVA with Sidak’s multiple comparisons test, Fig 6D), possibly due to the lower basal level. Comparing PTX-treated with saline-treated, cilostamide and rolipram together increased cAMP levels ~107% more in WT, ~28% more in AC5KO (P = 0.056, unpaired t-test, Fig 6D) and to the same extent in AC6KO (Fig 6D).

**Fig 7:**
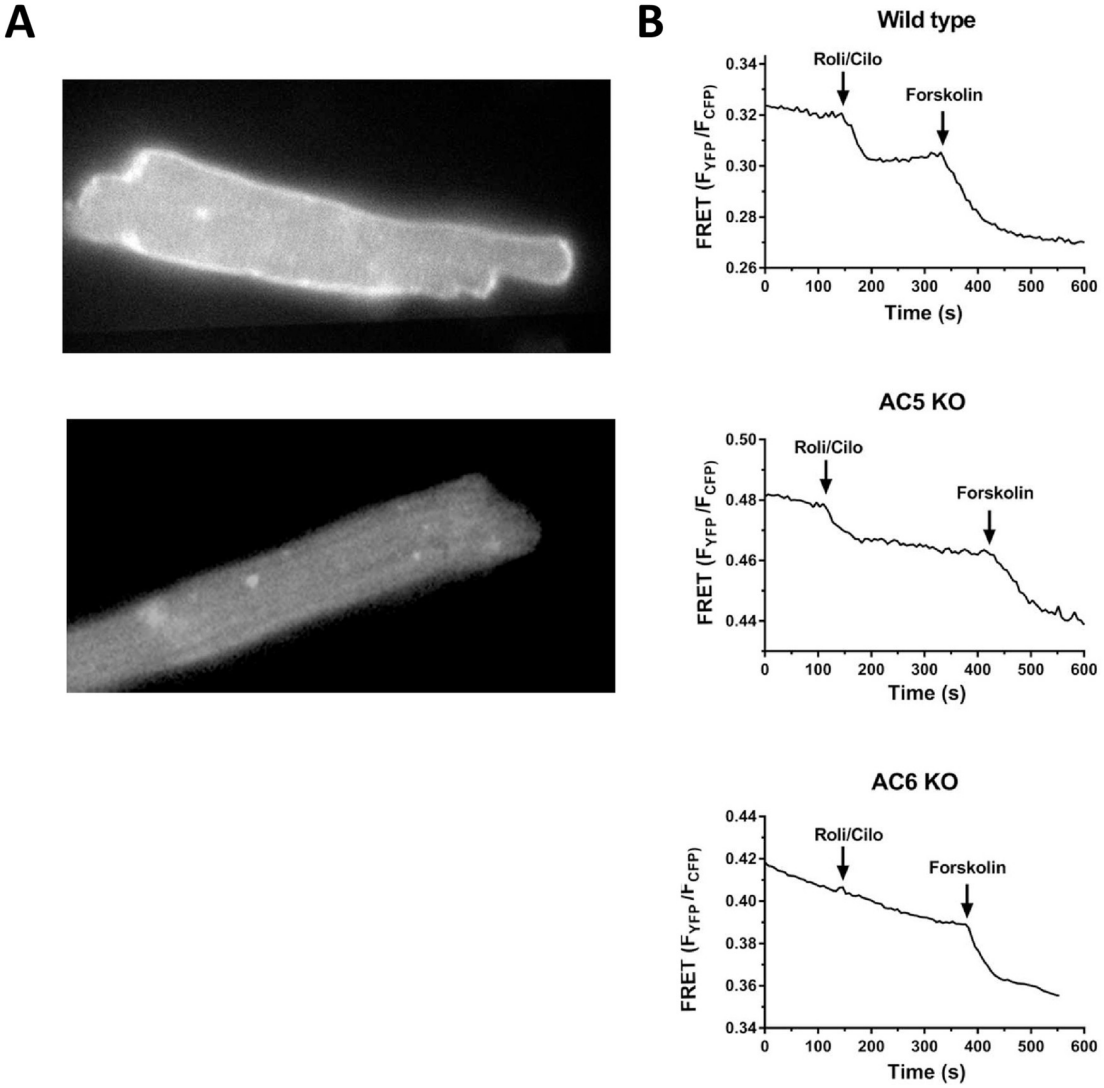
Increase in cAMP at the plasma membrane evoked by simultaneous inhibition of PDE3 and PDE4 only in WT and AC5KO. (A) Representative cardiomyocytes excited at 500±10 nm expressing (top) and not expressing (bottom) the pmEPAC1 sensor. Note the stronger signal on the plasma membrane of the top cell. (B) Representative traces displaying FRET detection of cAMP at the plasma membrane of PTX-treated cardiomyocytes after simultaneous inhibition of PDE3 (cilostamide, 1μM) and PDE4 (rolipram, 10 μM) in wild type (top), AC5KO (middle) and AC6KO (bottom). Isolated cardiomyocytes were incubated for 3 hours with 1 μg/ml PTX or saline (data not shown) of equal volume in the incubation media (1.2 ml reaction volume). FRET experiments were conducted in the presence of 1 μM timolol to block βARs.

Since the increase in cAMP levels of muscle strips was relatively modest, we crossbred the WT, AC5KO and AC6KO mice with mice expressing the pmEPAC1 sensor to detect cAMP levels near the plasma membrane [29]. As shown in Fig 7A, a higher fluorescence signal (bright white) was highly localized to the plasma membrane of cardiomyocytes expressing pmEPAC1 compared to mice without. All of the FRET data are summarized in Table 2. In PTX-treated WT cardiomyocytes, twelve of fourteen cells responded to cilostamide and rolipram with an average percent change in (F_YFP_/F_CFP_) of 4.62±0.65 from baseline (Fig 7B; Table 2A). The average change in response to forskolin was 9.09±1.27. In PTX-treated AC5KO cardiomyocytes, six of seven cells responded to cilostamide and rolipram with an average percent change in (F_YFP_/F_CFP_) of 5.24±1.1 from baseline (Fig 7B; Table 2A). The average change in response to forskolin was 9.27±1.06. In contrast, in PTX-treated AC6KO cardiomyocytes, only one of fourteen cells responded to cilostamide and rolipram. The response ratio in AC6KO is lower than the response ratios for WT and AC5KO (Table 2A). Nevertheless, the average forskolin FRET response in AC6KO was similar to WT and AC5KO (Table 2A). In non PTX-treated cardiomyocytes, we observed a response to cilostamide and rolipram only in WT (one of ten cells) but not in AC5KO (zero of six cells) or AC6KO (zero of eleven cells). There were no differences in the forskolin or total FRET responses between treatment (PTX) or group (mouse strain, Table 2B). Taken together, these data are consistent with the functional data indicating AC6 is necessary to increase cAMP levels and to elicit an increase in contractility after inhibition of G_i_, PDE3 and PDE4.

**Table 2:** FRET response after simultaneous inhibition of PDE3 (1μM cilostamide) and PDE4 (rolipram, 10 μM) followed by the response to forskolin (100 μM) in cardiomyocytes from WT, AC5 KO and AC6 KO mice expressing the pmEPAC1 sensor.

**A**			
**PTX-treated**	**Wild type (n = 9)**	**AC5 KO (n = 5)**	**AC6 KO (n = 9)**
**Number of cells tested**	14	7	14
**Number of cells responding to Cil/Rol**	12	6	1
**Response ratio**	0.86*	0.86**	0.07
**Cil/Rol FRET (F**_**YFP**_**/F**_**CFP**_**) (% change vs. baseline)**	4.62±0.65	5.24±1.10	1.37
**Forskolin FRET (F**_**YFP**_**/F**_**CFP**_**) (% change vs. Cil/Rol)**	9.09±1.27	9.27±1.06	9.77±2.2
**Total FRET (F**_**YFP**_**/F**_**CFP**_**) (% change after forskolin vs. baseline)**	12.64±1.42	13.38±1.43	9.88±1.97
**Cil/Rol FRET (F**_**YFP**_**/F**_**CFP**_**) (% of total FRET)**	36.7±5.0	36.9±6.2	37.2^a^
**B**			
**Non PTX-treated**	**Wild type (n = 9)**	**AC5 KO (n = 4)**	**AC6 KO (n = 8)**
**Number of cells tested**	10	6	11
**Number of cells responding to Cil/Rol**	1	0	0
**Cil/Rol FRET (F**_**YFP**_**/F**_**CFP**_**) (% change vs. baseline)**	4.20	NA	NA
**Forskolin FRET (F**_**YFP**_**/F**_**CFP**_**) (% change vs. Cil/Rol)**	10.73±1.08	11.34±0.91	7.77±0.88
**Total FRET (F**_**YFP**_**/F**_**CFP**_**) (% change after forskolin vs. baseline)**	11.10±1.20	11.34±0.91	7.77±0.88
**Cil/Rol FRET (F**_**YFP**_**/F**_**CFP**_**) (% of total FRET)**	26.1^a^	NA	NA

Isolated cardiomyocytes were incubated for 3 h with 1 μg/ml PTX (A) or without PTX (B). Experiments were conducted in the presence of 1 μM timolol to block βARs. Data are presented as the percentage change (reduction) in FRET signal (F_YFP_/F_CFP_) compared to the previous condition, cilostamide and rolipram compared to baseline (third row), forskolin compared to the cilostamide and rolipram (fourth row). Total FRET is the percentage change in FRET signal after forskolin compared to baseline (fifth row). The sixth row is the Cil/Rol FRET reported as a percentage of total FRET signal. Data are mean±SEM, number of mice, (n) is given in brackets.

*P = 0.0001,

**P = 0.0009,

Fisher’s exact test.

^a^Since only one cell responded to Cil/Rol, this value was calculated from the single cell data only, not the group data.

## Discussion

There is a need to better understand the physiological and pathological roles of the different AC subtypes and their regulatory mechanisms in the heart. This will help answering questions such as why do cardiomyocytes express multiple isoforms of AC and why is AC6 overexpression beneficial [30, 31], whereas AC5 overexpression is detrimental [32] in different animal models of heart failure. Functional differences between AC5 and AC6 have not been investigated until recently due to the lack of isoform-specific antibodies and the relatively low amounts of AC expressed at the cell membrane (an approximated ratio of one βAR: two hundred G-proteins: three ACs [33]). In this study, we took advantage of WT, AC5KO and AC6KO mice to determine if receptor-independent G_i_ regulation of AC activity is subtype selective. Our data indicate that G_i_ regulates receptor-independent AC6 activity, but not AC5 activity, in a cAMP-dependent signaling compartment that regulates contractility in mouse ventricle. These data are consistent with the hypothesis that β_1_AR-mediated inotropic responses are mediated primarily through AC6 signaling. In support, Timofeyev et al. [16], reported that the β_1_AR can enhance L-type calcium current (I_Ca,L_) via both the AC5 and AC6 isoform [16] in mouse heart. However, β_1_AR enhancement of I_Ca,L_ through the AC5 isoform (in AC6KO) required the blockade of both PDE3 and PDE4 [16]. In these same ACKO mice, we recently reported data that indicated there may be two distinct populations of β_1_ARs distinguished by coupling to either AC5 or AC6 [34]. The data of Cosson et al. [34] also suggested that tonic G_i_ inhibition occurs only upon AC6, not AC5. These findings are consistent with the current study where both the FRET response (increase in cAMP signaling) and an inotropic response were dependent upon AC6 but not AC5. Taken together, these three studies indicate that the β_1_AR-mediated inotropic response in WT mouse ventricle is primarily, if not exclusively, mediated through AC6 activation, implicating AC6 in the so-called “contractile compartment”.

### G_i_ regulates AC6 in the contractile compartment

We previously reported that simultaneous inhibition of PDE3 and PDE4 evokes a large cAMP-dependent inotropic and lusitropic response in rat LV only after G_i_ inactivation with PTX [19, 20]. In addition, this effect of PTX did not result from removing constitutive receptor activation of G_i_, since neither the muscarinic inverse agonist atropine nor the non-selective adenosine-receptor inverse agonist CGS 15943 [35] alone or in combination increased contractile force or cAMP accumulation in the presence of simultaneous inhibition of PDE3 and PDE4 [19]. We interpret these findings to indicate that G_i_ exerts an intrinsic inhibitory activity upon AC independent of G_i_-coupled receptor stimulation. In this study, combined PDE3 and PDE4 inhibition in PTX-treated WT mouse LV produced similar results consistent with the rat data suggesting that receptor-independent intrinsic G_i_ inhibition of AC is a cross-species phenomenon. Further, combined PDE3 and PDE4 inhibition also evoked an inotropic response in PTX-treated AC5KO mice, but not in AC6KO mice (Fig 3B and 3C; Fig 5A). These data have several important implications: they indicate that G_i_ has an important role in regulating receptor-independent AC6 activity and that AC6 is localized to a signaling compartment that regulates contractile function. The findings that PDE3 and PDE4 inhibition in PTX-treated AC6KO evoked no change in either cAMP levels at the plasma membrane or contractility (no inotropic or lusitropic response observed) infers that AC5 is either insensitive to G_i_ inhibition and/or it is localized to a signaling compartment without the ability to regulate contractile function. In addition, that a decrease in relaxation time (Fig 5B) and an increase in–(dP/dt)_max_ (Table 1) with an earlier decline in pressure (Fig 2D and 2F) accompanied the inotropic response to PDE3 and PDE4 inhibition in PTX-treated WT and AC5KO is also consistent with these responses to PDE inhibition being cAMP-dependent.

### G_i_ likely regulates constitutive β_1_AR activity through inhibition of AC6

The finding that receptor-independent G_i_ regulation of AC6 likely differs from AC5 is consistent with accumulating data indicating that the two isoforms are differentially expressed and regulated. Although AC5 and AC6 share high amino acid sequence homology, particularly in the C1 and C2 regions that form the catalytic unit of the enzyme, the sequences are more divergent at important regulatory regions such as the N-terminus, the C1b region and the fourth intracellular loop [5]. AC6 is closely associated with G_i_, with the N-terminus and C1a region being important regulatory sites for Gα_i_ [36]. Interestingly, AC6 is primarily associated with the β_1_AR at the plasma membrane of the cardiomyocyte [16]. The β_1_AR has high constitutive activity, leading to an agonist-independent cAMP increase which is restrained by PDE4D8 degradation of cAMP [37]. Our results indicate that this β_1_AR-mediated cAMP increase is also restrained by constitutive G_i_ inhibition of AC6 which accompanied the reduced functional response in AC6KO (compare Fig 3C vs. 3A and 3B; Fig 3F vs. 3D and 3E; Fig 5A and 5B). Possibly, constitutive G_i_ inhibition provides another mechanism for maintaining low basal cAMP levels in the absence of agonist in a compartment otherwise highly dominated by βAR constitutive receptor activation.

We have previously found that the potency of noradrenaline at the β_1_AR (in the presence of a selective β_2_AR antagonist and inverse agonist) was increased approximately ten-fold by PTX pre-treatment in the rat [18]. Although G_i_ does not directly participate in the β_1_AR signaling pathway [38, 39], G_i_ does constitutively inhibit AC6, since AC6 is required for the PTX-mediated effects upon both signaling and contractility (Fig 3A–3C; Figs 5A and 6D). The finding that AC6 was also necessary for PTX to increase noradrenaline potency [34], further supports that AC6 is the primary AC associated with the β_1_AR to regulate contractility. After PTX treatment, AC6 is relieved from tonic G_i_ inhibition, allowing more efficient signal transduction from β_1_ARs at lower ligand concentrations. This results in increased agonist potency and in reaching the maximal response at lower concentrations of agonist since the maximal response remains unchanged in PTX- and saline-treated heart [18].

In contrast, AC5 is proposed to be in a different functional compartment being located in the t-tubules through its caveolin-binding sequence in the N-terminus [16]. The N-terminus is also important for the formation of a preformed complex between AC5 and the inactive Gα_s_ heterotrimer [40], providing close association between AC5 and G_s_. In the t-tubules, AC5 is primarily associated with β_2_ARs and to a lesser extent β_1_ARs [16]. This compartment is tightly regulated by the recruitment of a β-arrestin-PDE4D5 complex upon β_2_AR activation [37]. That we did not observe an inotropic response (Figs 3C and 5A) or change in FRET to PDE3/4 inhibition in PTX-treated AC6KO mice (Fig 7B; Table 2), suggests that AC5 is not as sensitive to constitutively active G_i_ activity as AC6. Interestingly, we have observed that PTX treatment increased the maximum inotropic response evoked by adrenaline through activation of the β_2_AR by ~88% (in the presence of selective β_1_AR inverse agonist) without changing adrenaline potency [18]. In contrast to the β_1_AR, this likely reflects a removal of dual control by both G_s_ and G_i_ upon β_2_AR activation [38, 39], whereby removal of the G_i_ inhibitory restraint upon AC would allow for a larger increase in cAMP at all agonist concentrations including those that elicit the maximal response. That PTX treatment only increased efficacy but not potency of the β_2_AR stimulation is consistent with this hypothesis [18].

Taking into consideration data from prior studies together with the current findings suggests that G_i_ exerts a receptor-independent inhibition upon AC6. In addition, the data are consistent with the idea that AC6 resides in a compartment likely with β_1_ARs that can readily regulate contractile function as opposed to the AC5 isoform which is primarily localized with β_2_ARs [16]. It is reasonable to assume that our data highlight the propensity of AC to be spontaneously active and that inhibitory mechanisms such as G_i_ and PDEs are required to maintain low basal cAMP levels. In this respect, PTX allowed us to visualize the role of G_i_ to limit both receptor-dependent and independent spontaneous inactivation of AC.

## Abbreviations

AC: adenylyl cyclase
AC5KO: adenylyl cyclase type 5 knockout
AC6KO: adenylyl cyclase type 6 knockout
BDM: 2,3-butanedione monoxime
Cil: cilostamide
dF/dt: development of force over time
dP/dt: development of pressure over time
G_i_: inhibitory G protein
G_s_: stimulatory G protein
LV: left ventricle
PDE: phosphodiesterase
PKA: protein kinase A
PTX: pertussis toxin
Rol: rolipram
TCA: trichloroacetic acid
WT: wildtype
